# Sequential change in olfaction and (non) motor symptoms: the difference between anosmia and non-anosmia in Parkinson’s disease

**DOI:** 10.3389/fnagi.2023.1213977

**Published:** 2023-07-18

**Authors:** Ting-Chun Fang, Yu-Shan Tsai, Ming-Hong Chang

**Affiliations:** ^1^Department of Neurology, Taichung Veterans General Hospital, Neurological Institute, Taichung, Taiwan; ^2^Department of Post-Baccalaureate Medicine, College of Medicine, National Chung Hsing University, Taichung, Taiwan; ^3^Brain and Neuroscience Research Center, College of Medicine, National Chung Hsing University, Taichung, Taiwan

**Keywords:** Parkinson’s disease, olfactory dysfunction, UPSIT, MDS-UPDRS, equivalence dose of daily levodopa, cognition, depression

## Abstract

**Introduction:**

Hyposmia is a common prodrome in patients with Parkinson’s disease (PD). This study investigates whether olfactory changes in PD differ according to the degree of olfactory dysfunction and whether there are changes in motor and non-motor symptoms.

**Methods:**

The 129 subjects with PD were divided into two groups: anosmia and non-anosmia. All cases were reassessed within 1–3 years after the initial assessment. The assessment included the MDS-Unified PD Rating Scale (MDS-UPDRS), the University of Pennsylvania Smell Identification Test (UPSIT), Beck’s Depression Inventory-II (BDI-II), Montreal Cognitive Assessment (MoCA), and equivalence dose of daily levodopa (LEDD). The generalized estimating equation (GEE) model with an exchangeable correlation structure was used to analyze the change in baseline and follow-up tracking and the disparity in change between these two groups.

**Results:**

The anosmia group was older and had a longer disease duration than the non-anosmia group. There was a significant decrease in UPSIT after follow-up in the non-anosmia group (β = −3.62, *p* < 0.001) and a significant difference in the change between the two groups (group-by-time effect, β = 4.03, *p* < 0.001). In the third part of the UPDRS motor scores, there was a tendency to increase the score in the non-anosmia group compared to the anosmia group (group-by-time effect, β = −4.2, *p* < 0.038). There was no significant difference in the group-by-time effect for UPDRS total score, LEDD, BDI-II, and MoCA scores.

**Discussion:**

In conclusion, this study found that olfactory sensation may still regress in PD with a shorter disease course without anosmia, but it remains stable in the anosmia group. Such a decline in olfaction may not be related to cognitive status but may be associated with motor progression.

## 1. Introduction

Parkinson’s disease (PD) is the second most common neurodegenerative disease, with a prevalence of around 1.4–3.0 per thousand in Taiwan which increases with aging (Liu et al., 2016a,b). In addition to motor symptoms such as bradykinesia, tremor, and rigidity, non-motor symptoms contribute to poor quality of life in patients with PD (Rodríguez-Violante et al., 2015; Tibar et al., 2018; Santos Garcia et al., 2019). Some non-motor symptoms appear before motor symptoms, known as prodromal non-motor symptoms of PD (Poewe et al., 2017). Olfactory dysfunction, constipation, depression, and rapid eye movement (REM) sleep behavior disorder (RBD) can represent prodromal symptoms.

The Braak staging system explains prodromal symptoms because alpha-synuclein aggregates, a pathological hallmark of PD, are initially found in the olfactory bulb and the dorsal motor nucleus of the vagus (Braak et al., 2003). One route of propagation of alpha-synuclein inclusion in the dual-hit hypothesis starts from the enteric nervous system with the gut to brain spreading. This route is suggested to be associated with the involvement of the autonomic nervous system and premotor RBD, naming the body-first subtype. The other route of alpha-synuclein pathology starts from the olfactory bulb and anterior olfactory nucleus and spreads to adjacent areas such as the olfactory tubercle, piriform cortex, periamygdaloid cortex, and entorhinal cortex. However, the evidence of entry via the olfactory pathway is still controversial because no advanced lesions are found in non-olfactory cortical areas (Braak et al., 2003; Horsager et al., 2020). In a PD mouse model, RBD-like behavior occurred earlier than hyposmia, which correlates with the finding in humans that PD patients with RBD were more hyposmic than PD patients without RBD (Taguchi et al., 2020). These findings suggest that the ascending pathway of the brainstem may predominate in the spread of alpha-synuclein, despite the initial deposition in the olfactory bulb (Braak et al., 2003; Horsager et al., 2020).

However, hyposmia is still one of the common non-motor symptoms in PD related to Lewy body pathology in the olfactory system (Haehner et al., 2009; Rodríguez-Violante et al., 2017). As the disease progresses, Lewy body pathology increases in the olfactory system, but most studies show inconsistent results in the relationship between hyposmia and disease severity (Berendse et al., 2011; Yoo et al., 2020). Few studies discuss the association between the duration of the disease and olfactory dysfunction, and most of them did not show an obvious correlation, and even the results of some longitudinal studies are inconsistent (Ercoli et al., 2022). Due to the ambiguous relationship between olfactory dysfunction and disease duration, this study aims to investigate the longitudinal change of olfactory function in PD patients based on their degree of olfactory dysfunction. Given this uncertainty, we also conducted a comprehensive analysis of disease severity, medication usage, cognitive function, and depression during the longitudinal follow-up period to provide a more comprehensive comparison.

## 2. Materials and methods

### 2.1. Participants

Participants were recruited from the outpatient clinic at Taichung Veteran General Hospital from 2017. Subjects were selected on the basis of International Parkinson and MDS Clinical Diagnostic Criteria for Parkinson’s disease. At the first visit (T0), all subjects received a complete survey that included the MDS-Unified Parkinson’s Disease Rating Scale (MDS-UPDRS), the University of Pennsylvania Smell Identification Test (UPSIT), Beck’s Depression Inventory-II (BDI-II), and Montreal’s Cognitive Assessment (MoCA). Follow-up (T1) was conducted for these patients with PD in 1–3 years after the first visit, and a comprehensive survey was also conducted including MDS-UPDRS, UPDIT, BDI, and MoCA. The equivalent dose of daily levodopa was also calculated on the date of the first visit and the follow-up, respectively. Individuals were excluded if they did not meet the MDS clinical diagnostic criteria for Parkinson’s disease during follow-up or could not complete the questionnaire. Those who had upper respiratory tract infection and sinonasal disease which might affect olfaction were also excluded. Written informed consent was obtained from all participants. This study was approved by Taichung Veterans General Hospital Institutional Review Board/Ethics Committee (No. CE22189B). All methods were performed in accordance with the Declaration of Helsinki guidelines and hospital regulations.

### 2.2. Variables

The olfactory function was evaluated with the validated Taiwanese version of UPSIT, an odor identification (Jiang et al., 2010). The total score was 40 in this test and the cutoff value of total anosmia was less than 19. Considering that the mean UPSIT score is 17–20 in PD patients which is close to the cutoff value 19 of anomia in UPSIT, we divided subjects into two groups, anosmia and non-anosmia based on the UPSIT score at the first visit to represent the characteristics of profound olfactory deficit or milder symptom in PD, respectively (Doty, 2001, 2012; Picillo et al., 2014; Lawton et al., 2016). Non-motor symptoms of PD were also assessed. For cognition, we used MoCA due to its validation for assessing global cognitive abilities in PD (Litvan et al., 2012). BDI-II was used for mood investigation (Beck et al., 1996). Regarding the severity of motor symptoms in PD, the part 3 score of MDS-UPDRS (UPDRS 3) and the equivalent dose of daily levodopa (LEDD) were used to determine the severity of motor symptoms (Goetz et al., 2008). The total score of MDS-UPDRS (UPDRS T) was used to represent the disease burden of PD. Scores of MDS-UPDRS Part 1 and Part 2 were used to represent the non-motor and motor experiences of daily living, and Part 4 was used for motor complication. To determine the motor subtypes, we utilized 11 items (2.10, 3.15–3.18) for tremor and five items (2.12, 2.13, 3.10–3.12) for postural instability/gait difficulty (PIGD) from the MDS-UPDRS. The ratio of mean tremor scores to the mean PIGD scores was employed to define the following subtypes: (1) tremor subtype with a ratio ≥ 1.15 and (2) PIGD subtype with a ratio ≤ 0.90 (Stebbins et al., 2013).

### 2.3. Statistical analysis

Baseline clinical characteristics between the anosmia and non-anosmia groups were compared by using chi-square test for binary variables. UPSIT, MoCA, BDI-II, MDS UPDRS scores, and LEDD scores were analyzed as continuous variables. For continuous variables that follow a normal distribution, Student *t*-tests were used for analysis. For variables that do not follow a normal distribution, non-parametric Mann–Whitney U tests were used for analysis. Multiple linear regression adjusted for age, gender and disease was carried out to analyze the relationship between UPSIT and each variable including MoCA, BDI-II, UPDRS 3, UPDRS T, and LEDD scores at baseline. Generalized estimating equation (GEE) model with an exchangeable correlation structure, was used to assess the change of longitudinal data, including MoCA, BDI-II, UPDRS 3, UPDRS T, and LEDD, between the anosmia and non-anosmia groups at T1 compared with T0.

All tests were with a statistical significance level of *p* < 0.05 and were reported with 95% confidence intervals (CIs). Data analysis was performed with SPSS software (IBM Corporation, Armonk, New York, NY, USA).

## 3. Results

### 3.1. Demographic data

A total of 129 participants were enrolled in this study. Table 1 shows that the anosmia and non-anosmia groups comprised 73 and 56 subjects, respectively. At baseline, the anosmia group was older than the non-anosmia group (66.65 vs. 63.21, *p* = 0.032) and had a longer disease duration (4.89 years vs. 3.27, *p* = 0.033). The group with anosmia also demonstrated higher scores on UPDRST and UPDRS3, but exhibited lower scores on the MoCA. However, no significant differences were found between the two groups regarding gender, follow-up interval, motor subtypes, scores of UPDRS1, 2, and 4, LEDD and BDI scores. After the follow-up for UPSIT re-evaluation, it was observed that 20 patients from the non-anosmia group at the first visit had developed anosmia, accounting for 35.7% of the non-anosmia group. Conversely, seven patients from the anosmia group had transitioned to non-anosmia. Eventually, the anosmia and non-anosmia groups comprised 86 and 43 subjects, respectively.

**TABLE 1:** Characteristics of the participants at baseline.

Characteristic	Anosmia (*n* = 73)	Non-anosmia (*n* = 56)	*P*-value
Age, mean (SD), y	66.65 (8.63)	63.21 (9.33)	0.032*
Gender (%)			
Male	39 (53.5)	37 (66.1)	0.148
Female	34 (46.5)	19 (33.9)	
Disease duration, mean (SD), y	4.89 (5.07)	3.27 (3.46)	0.033*
Follow-up interval, mean (SD), y	1.52 (0.63)	1.65 (0.73)	0.288
UPDRST^†^	51.0 (37.0, 66.0)	43.0 (28.2, 53.0)	0.007*
UPDRS1^†^	9.0 (5.0, 14.0)	8.0 (5.0, 11.0)	0.378
UPDRS2^†^	8.0 (3.0, 13.5)	7.0 (3.2, 10.7)	0.211
UPDRS3^†^	32.0 (23.0, 41.0)	25.5 (17.0, 34.7)	0.002*
UPDRS4^†^	0 (0, 1.0)	0 (0, 0.7)	0.843
Motor subtype (%)			0.907
PIGD	34 (46.6)	24 (42.9)	
Tremor	25 (34.2)	21 (37.5)	
Indeterminate	14 (19.2)	11 (19.6)	
LEDD^†^	474.0 (201.8, 787.5)	377.5 (140.6, 637.5)	0.090
MoCA^†^	26.0 (21.5, 28.0)	27.0 (25.0, 29.0)	0.016*
BDI^†^	10.0 (4.0, 17.0)	8.5 (4.0, 14.5)	0.447

y, years; SD, standard deviation; UPDRS, MDS-UPDRS; LEDD, equivalent dose of daily levodopa; MoCA, Montreal’s Cognitive Assessment; BDI-II, Beck’s Depression Inventory-II; PIGD, postural instability/gait difficulty. ^†^Analyzed by non-parametric Mann–Whitney U-tests, and presented with median (1st and 3rd quartile). *Significance, *p* < 0.05.

### 3.2. Correlations between UPSIT and clinical features at baseline

The UPSIT scores of all participants at baseline were significantly correlated with MoCA (β = 0.14, *p* = 0.015), UPDRS 3 (β = −0.67, *p* = 0.001), and UPDRS T (β = −0.84, *p* = 0.007), after adjusting for age, gender, and disease duration. However, no significant correlations were found between UPSIT and BDI or LEDD.

### 3.3. Change in olfactory identification in anosmia/non-anosmia groups

**FIGURE 1:**
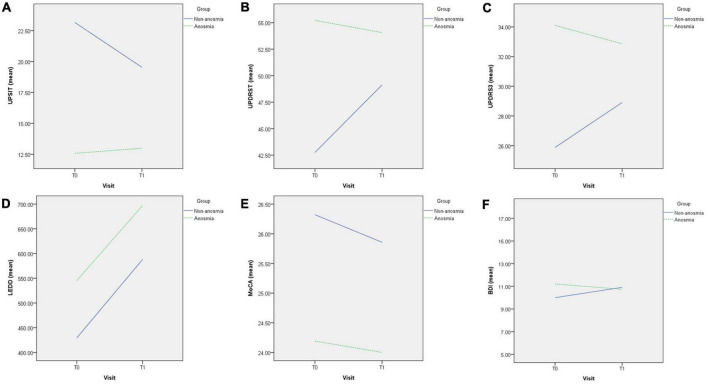
Difference in changes between non-anosmia and anosmia group in UPSIT **(A)**, UPDRST **(B)**, UPDRS3 **(C)**, LEDD **(D)**, MoCA **(E)**, and BDI-II **(F)** during the follow-up. T0, first visit; T1, follow-up visit; UPSIT, University of Pennsylvania Smell Identification Test; UPDRST, total score of MDS-UPDRS; UPDRS3, part 3 score of MDS-UPDRS; LEDD, equivalent dose of daily levodopa; MoCA, Montreal’s Cognitive Assessment; BDI-II, Beck’s Depression Inventory-II.

In the GEE analysis (Table 2), a significant group effect revealed a lower UPSIT score in the anosmia group (β = −10.58, *p* < 0.001). The time effect was significant in the non-anosmia group (β = −3.62, *p* < 0.001) but not in the anosmia group. The group-by-time effect was also significant (β = 4.03, *p* < 0.001), indicating that the UPSIT score remained stable in the anosmia group but decreased significantly in the non-anosmia group (Figure 1A). These results remained significant after adjusting for age, gender, and disease duration (Table 3).

**TABLE 2:** Generalized estimating equation analysis for the comparison of outcomes.

	Mean (SD)		Group effect (anosmia vs. non-anosmia)		Time effect, anosmia (T1 vs. T0)		Time effect, non-anosmia (T1 vs. T0)		Group × time effect	
**Outcome**	**Anosmia**	**Non-anosmia**	***B***	***P*-value**	***B***	***P*-value**	***B***	***P*-value**	***B***	***P*-value**
**UPSIT**										
T0	12.57 (4.45)	23.16 (3.46)	−10.58	<0.001*	NA	NA	NA	NA	NA	NA
T1	12.98 (4.75)	19.53 (6.29)			0.41	0.55	−3.62	<0.001*	4.03	<0.001*
**LEDD**										
T0	545.39 (389.33)	429.43 (325.62)	115.95	0.064	NA	NA	NA	NA	NA	NA
T1	697.40 (443.47)	588.23 (360.69)			152.01	<0.001*	158.79	<0.001*	−6.78	0.879
**MoCA**										
T0	24.19 (5.36)	26.32 (3.57)	−2.13	0.007*	NA	NA	NA	NA	NA	NA
T1	24.00 (5.22)	25.85 (3.88)			−0.19	0.684	−0.46	0.183	0.27	0.642
**BDI-II**										
T0	11.20 (8.63)	10.00 (8.23)	1.2	0.416	NA	NA	NA	NA	NA	NA
T1	10.73 (7.89)	10.90 (9.82)			−0.46	0.682	0.83	0.462	−1.29	0.418
**UPDRS3**										
T0	34.10 (14.82)	25.87 (12.23)	8.23	<0.001*	NA	NA	NA	NA	NA	NA
T1	32.86 (13.64)	28.91 (9.54)			−1.24	0.425	3.03	0.022*	−4.28	0.037*
**UPDRST**										
T0	55.21 (25.71)	42.75 (18.66)	12.46	0.001*	NA	NA	NA	NA	NA	NA
T1	54.06 (24.00)	49.12 (21.29)			−1.15	0.683	6.37	0.014*	−7.52	0.049*

T0, first visit; T1, follow-up visit; SD, standard deviation; B, beta coefficient; UPSIT, University of Pennsylvania Smell Identification Test; LEDD, equivalent dose of daily levodopa; MoCA, Montreal’s Cognitive Assessment; BDI-II, Beck’s Depression Inventory-II; UPDRS3, part 3 score of MDS-UPDRS; UPDRST, total score of MDS-UPDRS. *Significance, *p* < 0.05.

**TABLE 3:** Generalized estimating equation analysis for the comparison of outcomes, adjusted for age, gender, and disease duration.

	Mean (SD)		Group effect (anosmia vs. non-anosmia)		Time effect, anosmia (T1 vs. T0)		Time effect, non-anosmia (T1 vs. T0)		Group × time effect	
**Outcome**	**Anosmia**	**Non-anosmia**	***B***	***P*-value**	***B***	***P*-value**	***B***	***P*-value**	***B***	***P*-value**
**UPSIT**										
T0	12.57 (4.45)	23.16 (3.46)	−10.39	<0.001*	NA	NA	NA	NA	NA	NA
T1	12.98 (4.75)	19.53 (6.29)			0.55	0.435	−3.48	<0.001*	4.03	<0.001*
**LEDD**										
T0	545.39 (389.33)	429.43 (325.62)	84.79	0.112	NA	NA	NA	NA	NA	NA
T1	697.40 (443.47)	588.23 (360.69)			116.9	0.001*	122.28	0.001*	−5.37	0.904
**MoCA**										
T0	24.19 (5.36)	26.32 (3.57)	−1.26	0.061	NA	NA	NA	NA	NA	NA
T1	24.00 (5.22)	25.85 (3.88)			−0.01	0.978	−0.23	0.53	0.21	0.711
**BDI-II**										
T0	11.20 (8.63)	10.00 (8.23)	0.47	0.751	NA	NA	NA	NA	NA	NA
T1	10.73 (7.89)	10.90 (9.82)			−0.8	0.491	0.42	0.712	0	0.447
**UPDRS3**										
T0	34.10 (14.82)	25.87 (12.23)	6.9	0.002*	NA	NA	NA	NA	NA	NA
T1	32.86 (13.64)	28.91 (9.54)			−1.9	0.239	2.32	0.095	−4.2	0.038*
**UPDRST**										
T0	55.21 (25.71)	42.75 (18.66)	8.52	0.015*	NA	NA	NA	NA	NA	NA
T1	54.06 (24.00)	49.12 (21.29)			−3.31	0.265	4.01	0.138	−7.33	0.054

T0, first visit; T1, follow-up visit; SD, standard deviation; B, beta coefficient; UPSIT, University of Pennsylvania Smell Identification Test; LEDD, equivalent dose of daily levodopa; MoCA, Montreal’s Cognitive Assessment; BDI-II, Beck’s Depression Inventory-II; UPDRS3, part 3 score of MDS-UPDRS; UPDRST, total score of MDS-UPDRS. *Significance, *p* < 0.05.

### 3.4. Change in motor symptoms in anosmia/non-anosmia groups

Significant group effects were found for UPDRS 3 and UPDRS T (Table 3), indicating higher scores in the anosmia group at baseline. Although there were trends of increasing UPDRS T and UPDRS 3 scores in the non-anosmia group at follow-up (Figures 1B, C and Table 2), the time effect lost its significance after adjustment (Table 3). However, the group-by-time effect for UPDRS 3 remained significant (β = −4.2, *p* < 0.038) after adjusting for age, gender, and disease duration (Table 3). The increase in UPDRS 3 score was much more pronounced in the non-anosmia group (Figure 1C).

Regarding LEDD, the time effects for both the anosmia group (β = 116.90, *p* = 0.001) and the non-anosmia group (β = 122.28, *p* = 0.001) were significant, but there was no significance in either group effect or group-by-time effect (Figure 1D and Table 3).

### 3.5. Change in cognition and mood in anosmia/non-anosmia groups

The MoCA score was lower in the anosmia group with a significant group effect (β = −2.13, *p* = 0.007) (Table 2), but the significance disappeared after adjusting for age, gender, and disease duration (Table 3). No significant effects for MoCA were found for time or group-by-time effects (Figure 1E and Table 3). Likewise, no significant effects were found for BDI in terms of group effect, time effect, or group-by-time effect (Figure 1F and Table 3).

## 4. Discussion

The present study demonstrated that the UPSIT score regressed in the non-anosmia group while remaining stable in the anosmia group. Notably, the non-anosmia group had a relatively short course of the disease in this study. Our findings are consistent with those of other longitudinal studies. For instance, Lewis et al. (2020) analyzed PD patients annually and found that UPSIT significantly decreased in early and middle-stage PD but not in later-stage PD with disease duration exceeding 5 years. Domellof et al. (2017) explored the UPSIT outcome with the interaction effect between the group (hyposmic/normosmic) and time, revealing that UPSIT deteriorated over time in the normosmic group while remaining stable in the hyposmic group. Meusel et al. (2010) showed a larger olfactory decline in the subgroup of patients with no severe initial olfactory deficit over 5 years of tracking. The patients with marked olfactory regression had an average disease duration of 2.3 years at the beginning of the visit.

Our results support these findings by indicating that the rate of olfactory decline with disease progression is more pronounced in patients without severe initial olfactory deficits, whereas the olfactory deficit remains relatively stable in patients with profound olfactory deficits. While olfactory impairment is considered a premotor feature of Parkinson’s disease (PD), it is important to note that the olfactory impairment may continue to progress even after motor symptoms have emerged until it reaches a point known as the “floor effect” in the current olfactory test (Fullard et al., 2017). This corresponds to the hypothesis proposed by Huisman et al. (2004) suggesting that dopaminergic neurons in the olfactory bulb, which act as possible suppressors in olfactory transmission, increase as a compensatory mechanism to the dopamine deficit in the basal ganglia. With disease progression, the decrease in olfactory bulb volume and the deposition of Lewy bodies in the olfactory bulb may neutralize such inhibitory changes, resulting in less significant olfactory degeneration (Herting et al., 2008). However, olfactory loss in PD may not be simply explained by imbalance of dopamine projection because the olfactory function involves several neurotransmitters such as acetylcholine, norepinephrine, serotonin and GABA (Doty, 2017). As olfactory dysfunction appears to be more closely associated with the body-first type of alpha-synuclein propagation, the pathology primarily affecting the dorsal motor nucleus of the vagus or brainstem may impact olfactory function through the development of alpha-synucleinopathy in the bilateral olfactory bulbs or other brainstem nuclei that project to the olfactory system (Borghammer, 2021). Some cross-sectional studies have shown that olfactory degeneration is unrelated to the disease’s course (Cavaco et al., 2015; Masala et al., 2018). Other longitudinal studies have also shown no significant change in olfaction over time in patients with PD (Doty et al., 1988; Muller et al., 2002; Herting et al., 2008; Campabadal et al., 2017; Fujio et al., 2020). Such different results may be related to different study designs, such as the number of patients enrolled, the characteristics of different patient groups, and so on. In our study, patients were divided into two groups, anosmia and non-anosmia, and the course of the disease differed between the two groups. Therefore, grouping patients according to the degree or duration of olfactory abnormalities may explain the discrepancies between the results of these studies.

Olfactory deterioration in patients with PD is thought to be associated with cognitive decline, and in particular, the accuracy of olfactory identification tests is often affected by cognitive decline (Laing and Doty, 2003). However, the results of this study showed that although the UPSIT scores of the non-anosmia group decreased after follow-up, there was no significant difference in the MoCA scores for the cognitive function component. This may suggest that while there is a significant association between hyposmia in PD patients and cognitive decline, the initial regression in olfactory identification is not solely attributed to cognitive decline. Other factors, such as Lewy body-related pathology in the peripheral and central olfactory organs or change in the balance of neurotransmitters, may play a role.

Regarding disease severity, although the association with olfactory abnormalities remains inconclusive, our study found a significant association between UPSIT and UPDRS T score and UPDRS 3 scores, in line with the results of other studies (Roos et al., 2019). Unlike the longitudinal study by He et al. (2020) which showed that olfactory abnormalities were predictive of disease progression, our study found no change in UPDRS T and UPDRS 3 score in the anosmia group during short-term follow-up, but there was a tendency for symptoms to progress in the non-anosmia group. These different results may be due to differences in the length of follow-up, patient subgroups, and analysis methods.

In addition, the worsening of Parkinson’s symptoms and olfaction in the non-anosmia group during the follow-up period may indirectly support the theory of Lewy body pathology between the brainstem and olfactory organs, as well as the influence of neurotransmitters such as dopamine. In the Braak staging system, Lewy body pathology was initially found in the olfactory bulb, but this lesion did not progress further, suggesting that a cascade of pathological changes from the brainstem upward is the main pathway (Braak et al., 2003). Horsager et al. (2020) proposed a body-first and brain-first model for the progression of PD pathology based on the presence or absence of RBD and the results of 123I-metaiodobenzylguanidine (MIBG) scintigraphy. The body-first model corresponds to the spreading pathway of the Braak staging system. In addition to autonomic-related prodrome and RBD, the body-first model has a faster progression of motor symptoms and earlier olfactory abnormalities than the brain-first model (Borghammer et al., 2021). These features of the body-first model may reflect the association between olfactory Lewy pathology and the caudo-rostral progression of Lewy pathology. However, olfactory Lewy pathology is not only related to caudo-rostral progression. Kok et al. (2021) found two features of olfactory Lewy pathology in the Vantaa85 + cohort: caudo-rostral progression and amygdala-based progression, corresponding to the body-first and brain-first models, respectively. This may also explain why not all patients in the non-anosmia group in our study turned to anosmia during follow-up and indicates that the severity and pathological changes of olfaction in PD are not a single pattern of progression. Further research with larger, more definitive patient classification, longer follow-up studies, and the inclusion of pathology and imaging is required to elucidate the relationship between olfaction and PD.

This study has some limitations. First, the follow-up period of 1–3 years and the single follow-up session may not have been sufficient to detect changes in clinical data over a longer period. However, changes in olfaction in patients with shorter disease duration and non-anosmia progressed within 3 years, while the severity of significant motor symptoms and cognitive function may require a longer follow-up period to observe a difference. Second, although we tried to exclude the possibility that olfactory tests were affected by diseases such as sinonasal disease or upper respiratory tract infection, which commonly affect the sense of smell, there are many other causes of olfactory abnormalities, including idiopathic causes (which may account for 18% of patients with olfactory abnormalities), that may affect test results (Temmel et al., 2002). Thirdly, for safety and the subjects’ preference, we used the On status UPDRS score for the assessment of motor symptoms and disease severity, and therefore, the assessment may be influenced by medication. Nevertheless, these patients are regularly followed up in the outpatient clinic, and the physician ensures that the patient’s medication dosage is adequate. We also analyzed LEDD, which showed that the non-anosmia group had a lower LEDD than the anosmia group, but there was no significant difference between the two. This indirectly implies that the non-anosmia group was not using fewer medications despite having a lower UPDRS score. Therefore, the effect of insufficient dosage of medication on the increasing UPDRS score in the non-anosmia group in this study may be subtle.

## 5. Conclusion

In conclusion, this study shows that olfactory sensation may still regress in Parkinson’s patients with a shorter course of the disease without anosmia, while it remains stable in the anosmia group. Such a decline in olfaction may not be related to cognitive status but may be associated with disease progression. Larger, long-term follow-up studies incorporating pathology and imaging analysis are needed to elucidate the underlying mechanisms.

## Data availability statement

The raw data supporting the conclusions of this article will be made available by the authors, without undue reservation.

## Ethics statement

The studies involving human participants were reviewed and approved by the Taichung Veterans General Hospital Institutional Review Board/Ethics Committee (No. CE22189B). The patients/participants provided their written informed consent to participate in this study.

## Author contributions

T-CF and M-HC conceptualized the project. T-CF and Y-ST performed the data acquisition and analysis. T-CF wrote the first draft of the manuscript. M-HC critically reviewed the manuscript. All authors contributed to writing and revising the manuscript.
